# Cost-effectiveness of smoke-free interventions: A systematic review

**DOI:** 10.18332/tpc/211801

**Published:** 2025-11-26

**Authors:** Kalin Werner, Tracy K. Lin, Rahaf H. Binshehah, Mohammed A. Shahin, Abdulmohsen H. Al-Zalabani, Mariam M. Hamza, Reem F. Alsukait, Volkan Cetinkaya, Taghreed Alghaith

**Affiliations:** 1 Institute for Health and Aging, Department of Social and Behavioral Sciences, University of California, San Francisco, San Francisco, United States; 2 Saudi Public Health Authority, Riyadh, Saudi Arabia; 3 Department of Family and Community Medicine, College of Medicine, Taibah University, Madinah, Saudi Arabia; 4 Health, Nutrition and Population Global Practice, The World Bank, Washington DC, United States

**Keywords:** public policy, economics, secondhand smoke

## Abstract

**INTRODUCTION:**

Tobacco use imposes substantial global economic costs – estimated at $1803 to $1899 billion annually (1.76–1.85% of global gross domestic product) – through healthcare expenditures and productivity losses. In 2019, it contributed to approximately 8.70 million deaths and 229.77 million disability-adjusted life years (DALYs); secondhand smoke exposure added 1.31 million deaths and 37.01 million DALYs. While tobacco control efforts continue, evidence on the economic impacts of interventions remains limited. This study systematically reviews the cost-effectiveness of smoke-free interventions to inform policy.

**METHODS:**

A systematic literature review was conducted following PRISMA guidelines and registered with PROSPERO (CRD42024521198). Searches conducted in January 2024 in PubMed, Web of Science, and Cochrane databases identified English-language economic evaluations of smoke-free interventions. Data were extracted using pre-defined checklists, and results were summarized using narrative synthesis. Studies were further assessed using the Consensus Health Economic Criteria (CHEC) and Consolidated Health Economic Evaluation Reporting Standards (CHEERS) tools. Studies scoring <60% on CHEERS were excluded.

**RESULTS:**

Of 639 articles, 9 met the inclusion criteria. Most used healthcare or government perspectives (56%), 33% adopting a societal perspective. Interventions included public (44%), workplace, and school-based smoking bans. Studies were conducted in Vietnam, the US, Indonesia, Tanzania, Estonia, Denmark, India, and EU countries, employing economic models such as Markov models (44%). Time horizons ranged from one year to lifetime. Incremental cost-effectiveness ratios (ICERs) ranged from $7 per DALY averted to $17056 per life year gained. Most interventions were found to be cost-effective or dominant.

**CONCLUSIONS:**

Public smoking bans consistently saved healthcare costs; workplace and school-based bans were also cost-effective, though context-dependent. This review suggests that smoking bans, particularly comprehensive smoke-free policies, should be prioritized by decision-makers as they demonstrate both health and economic benefits, proving to be cost-effective or even cost-saving public health interventions.

## INTRODUCTION

Tobacco use is a major global health and economic burden, leading to millions of deaths and significant costs^1^. The use of tobacco continues to be a leading contributor to disease burden and mortality globally, resulting in approximately 8.70 million deaths and 229.77 million disability-adjusted life years (DALYs) in 2019^2^. Furthermore, exposure to secondhand smoke leads to passive or involuntary smoking and results in harm to non-smokers. A large share of the population continues to be exposed to smoke emissions, with secondhand smoke exposure alone linked to over a million deaths and tens of millions of years healthy life lost in 2019^3,4^.

Both active smoking and secondhand smoke contribute to substantial economic losses, estimated at nearly 2% of global gross domestic product (GDP)^1^. Active tobacco use and passive smoke exposure attributed to tobacco (e.g. through secondhand smoking) contributed to the substantial economic costs associated with tobacco use, reaching an estimated total between $1803 and $1899 billion globally^1^. These costs include significant healthcare expenses for treating the diseases caused by tobacco use, as well as losses of human capital due to tobacco-attributable morbidity and mortality. More specifically, secondhand smoke contributes heavily to losses of labor and economic productivity^5^. In countries, secondhand smoke exposure accounts for anywhere between 10% and 20% of the total economic burden due to tobacco use costs^6,7^.

Recognizing the health and economic burden associated with tobacco use, the Framework Convention on Tobacco Control (FCTC) has been established. This is the first World Health Organization (WHO) treaty adopted under Article 19 of the WHO constitution, which requires signatory states to modify tobacco taxes and tariffs as well as formulate national policies to reduce the prevalence of smokers^8^. This health treaty is one of the first to coordinate and incentivize a reduction in tobacco use internationally. The FCTC’s efforts to reduce tobacco consumption globally since 2003 have resulted in increased international support and proliferation in anti-smoking legislative actions, including behavioral incentives through increased taxes and tariffs as well as regulation and laws mandating smoke-free environments^9^.

Systematic reviews and meta-analyses have documented that anti-smoking legislative actions in general reduce passive smoke exposure (i.e. secondhand smoke)^10,11^. Focusing specifically on the impact of smoke-free environments (also known as smoking bans) on health outcomes, there is increasing evidence to suggest that smoke-free environments are associated with improved health outcomes. For example, evidence indicated that smoking bans contribute to the reduction of smoking prevalence and can limit exposure to smoke and de-normalize the use of tobacco^12,13^.

Comparative evidence on the economic impact of smoke-free environments remains limited. There is a need for systematic reviews addressing the effect of smoke-free interventions on economic outcomes. As a result, the literature is somewhat fragmented, with several studies of limited rigor suggesting that regulations and mandates for smoke-free environments may negatively affect economic outcomes. On the other hand, analyses using more robust methods generally indicate that smoke-free interventions have little to no measurable impact on economic factors^14^.

The primary objective of this literature review is to systematically identify, summarize, and compare published evidence on the cost-effectiveness of smoke-free interventions. We aim to evaluate standardized outcomes (e.g. incremental cost-effectiveness ratio) and contribute to the discourse on the economic impact that smoke-free interventions have on health systems and societies. The findings may inform policymakers on the impact of mandates and laws that regulate tobacco use and contribute to decision-making processes.

## METHODS

We conducted a systematic literature review using the Preferred Reporting Items for Systematic Review and Meta-Analyses (PRISMA) guidelines^15^. The review was registered with PROSPERO (CRD42024521198). Three databases were searched (PubMed, Web of Science, Cochrane) in January 2024 for articles related to the cost-effectiveness of smoking bans. The following concepts were used in the development of search strings: 1) smoke-free; 2) smoking bans; and 3) economic evaluation. No filters were applied to our searches. Full search strings are available in Supplementary file Appendix 1. The reference lists of identified literature reviews were assessed to identify additional relevant studies for our analysis.

### Eligibility criteria

Studies had to be economic evaluations of public smoke-free interventions available in English. Inclusion criteria for studies to be entered in data extraction were: 1) an economic evaluation, and 2) an assessment of a smoke-free intervention or policy, and 3) in English. Our study included all degrees of smoking bans (e.g. full ban, partial ban, mandatory smoking ban). Literature reviews were not included in our study. To ensure a systematic comparison of studies with robust analysis and peer review, we excluded conference abstracts, posters, and protocols from our review. Inclusion and exclusion criteria are outlined in Table 1.

**Table 1:** PICOS inclusion and exclusion criteria

*Element*	*Inclusion criteria*	*Exclusion criteria*
**Population**	General population or subgroups (e.g. adults, adolescents, workers) exposed to tobacco smoke; populations targeted by smoke-free policies or interventions.	Studies limited to non-human populations, or populations in settings with limited autonomy such as incarcerated populations.
**Intervention**	Smoke-free interventions, such as: Public smoke-free laws/regulations (workplaces, hospitality venues, public spaces) Organizational policies (e.g. hospitals, schools, workplaces) Community-level smoke-free initiatives	Interventions not related to smoke-free environments (e.g. taxation, health warnings, cessation-only programs without a smoke-free component).
**Comparison**	No intervention (status quo) Pre-intervention baseline Alternative or less restrictive policies/interventions	Studies without a comparator group or with purely descriptive data (e.g. no cost-effectiveness analysis).
**Outcomes**	Economic evaluations reporting standardized outcomes, such as: - Incremental cost-effectiveness ratio (ICER) - Cost per QALY, DALY, or life-year gained	Studies not reporting economic outcomes (e.g. prevalence, attitudes, or health outcomes without cost-effectiveness/economic analysis).
**Study design**	Full economic evaluations (cost-effectiveness, cost-utility, cost-benefit, cost-consequence analyses) Model-based studies and empirical evaluations Peer-reviewed articles	Partial economic evaluations (cost-description only) Conference abstracts, posters, protocols, commentaries, editorials, letters Non peer-reviewed reports (unless part of grey literature strategy, if applicable)

Covidence software (www.covidence.org) was utilized by two independent reviewers, and conflicts were resolved through discussion. Duplicates were first removed using Covidence review software (Veritas Health Innovation, Melbourne, Australia), and then manually checked by the review team. Two reviewers independently screened each of the titles and abstracts for article relevance. After removing abstracts that did not meet the inclusion criteria, the full-text of the remaining articles were reviewed for eligibility. Conflicts were resolved through discussion among reviewers. All studies that passed full-text review were then assessed for risk of bias.

### Risk of bias assessment

Studies were assessed using the Consensus on Health Economic Criteria (CHEC) and Consolidated Health Economic Evaluation Reporting Standards (CHEERS) guidelines, with low-quality studies excluded. The quality of each of the included studies was assessed following the principles outlined in the CHEC list and using the CHEERS guidelines^16,17^. The CHEERS statement includes 27 checklist items across six categories, making it more comprehensive than the 19-item CHEC list. Checklist items range from a clear description of competing alternatives to the appropriate measurement of costs and value of outcomes, to inform how well an article has addressed the minimum quality elements of an economic evaluation. Following previously peer-reviewed and published systematic reviews that aim to only include studies that employed robust methodology and provided adequate information for analysis, we implemented a quality assessment cutoff score^18,19^. Studies that received fewer than 60% (16/27) of the CHEERS checklist were considered low-quality and excluded from our analysis.

### Data extraction and analysis

Key data from studies that met all inclusion criteria were abstracted by a single reviewer using a predefined 28-item checklist in the Covidence software, and later cross checked by a second reviewer. Relevant data to economic evaluations were extracted, including: model design, country of study, intervention, and comparator, characteristics of the modelled study population, perspective of analysis, time horizon, discount rate, currency and currency year, willingness to pay threshold and incremental cost-effectiveness ratio. Descriptive characteristics of included studies were tabulated based on details in the data extraction matrix. Included studies were then analyzed by theme through discussion and consensus amongst reviewers using narrative synthesis.

## RESULTS

**Figure 1:**
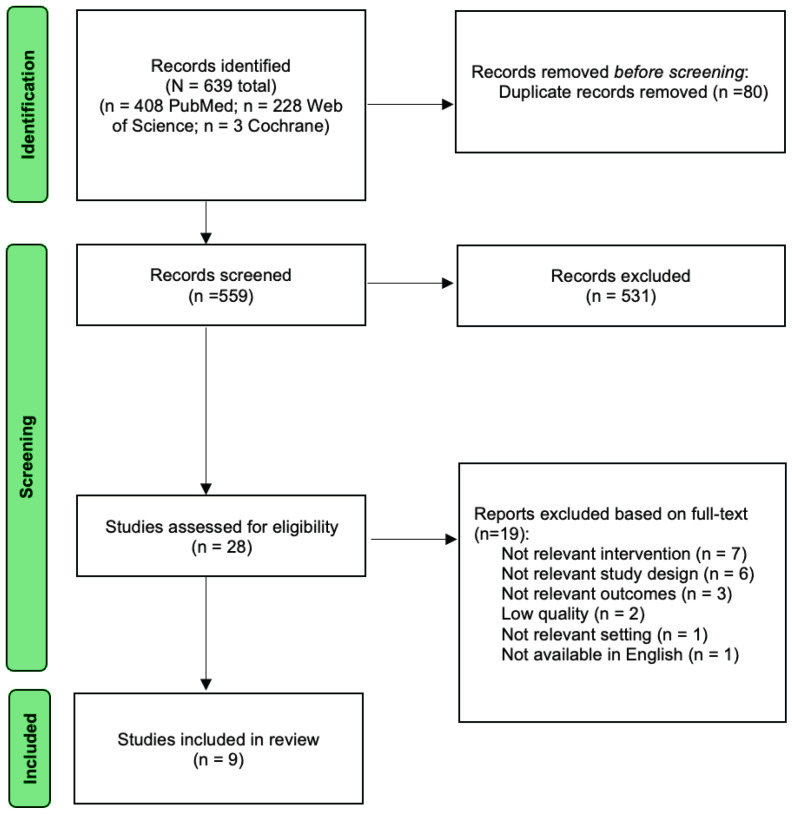
PRISMA flow diagram

Nine studies were included, assessing public, workplace, and school smoking bans in various countries. Initial database searches identified 639 articles. After the removal of 80 duplicates, we reviewed the titles and abstracts of 559 studies. Through the step of reviewing titles and abstracts, we excluded 531 titles and abstracts, resulting in 28 studies that were screened in the full-text review step. Of the 28 studies, 19 studies were excluded. We excluded seven studies that examined non-relevant interventions and six studies that did not incorporate economic evaluations. Concomitantly, we excluded three studies that did not report relevant outcomes and one study that did not take place in a study-relevant setting. Lastly, one single study was not available in English and was therefore excluded. Following our quality assessment, two studies were excluded due to a high risk of bias^20,21^. Results of the quality assessment can be found in Supplementary file Appendix 2. Our final analyses included 9 studies. Results are presented as a PRISMA flow diagram in Figure 1.

*Study characteristics*

Five descriptive characteristics were extracted from the included studies (n=9). Summaries are presented below with details presented in Supplementary file Appendix 3.

*Perspective chosen*

Most analyses were conducted from a healthcare and/or government perspective (56%, n=5)^22-26^. Three studies chose a more comprehensive societal perspective (33%)^27-29^. One study did not report the perspective used in the analyses^30^.

*Intervention and comparator*

Smoking bans in public places were the most commonly assessed intervention (44%, n=4)^22,26-28^. A single study assessed only workplace smoking bans^30^. Two studies assessed both public bans and workplace bans^23,24^. A single study reported on the cost-effectiveness of a school-based smoking ban^25^.

*Simulated population*

Studies assessed country-level populations or specific smoking populations within the country. One study focused on adolescents due to the school-based nature of the smoking-ban interventions^25^.

*Country and currency*

Models identified in our review were conducted across a variety of country contexts, including two studies conducted in Vietnam, and one study from each of the following: the US, Indonesia, Tanzania, Estonia, Denmark, India, and a collection of seven EU countries. Costs were commonly expressed in US$ (33%, n=3)^23,26,30^. The remaining studies were reported in local currencies including Euros^25^, Estonian Kroon (EEK)^29^, Danish Krone (DKK)^27^, Vietnamese Dong (VND)^22,24^, and Indian Rupees (INR)^28^.

*Cost estimations and effectiveness outcome measures*

The majority of studies only assessed direct costs (67%, n=6)^23-26,30^, while the remaining three considered both direct and indirect costs^27-29^. Sources of cost data were primarily from the literature or government database; although two studies used activity-based costing approaches to identify costs used in the model^22,25^. Five technical characteristics of the economic analyses were extracted from the included studies and are outlined in detail below. Further details can be found in Supplementary file Appendix 2.

*Modeling approach*

Markov model designs were used in the highest number of studies (67%, n=6)^22-27^. One study used a decision-analytic model approach^28^. Two studies did not specify the type of modeling approach used^29,30^, although one noted that the WHO cost-effectiveness modeling framework was followed^29^.

*Time horizon*

Included studies used time horizons that ranged from one year to a lifetime. The majority of the studies used a 10-year horizon (56%, n=5)^22,23,27,28^.

*Discounting*

The majority of studies used a 3% discount rate (78%, n=7)^22-24,26,28-30^ although two studies used a slightly higher 3.5% discount rate (22%, n=2)^25,27^.

*Threshold used*

Most authors followed the WHO recommended one time’s GDP per capita as the threshold at which interventions would be considered cost-effective in their analysis (78%, n=7)^22-26,28,29^. A single study used the standard US threshold of $50000/Quality adjusted life year (QALY)^30^.

*Sensitivity analyses*

Four of the included studies performed only deterministic sensitivity analyses^22,25,27,29^. Three studies only mentioned performing the more comprehensive probabilistic sensitivity analysis^23,24,30^, and two studies performed both deterministic and probabilistic sensitivity analyses^26,28^.

*Cost-effectiveness of interventions*

The incremental cost-effectiveness ratio captured in this review ranged from $7 per DALY averted to $17056 per life year gained. Four studies found that smoking bans dominated (i.e. the interventions were less costly and more effective than their comparator). A single study included an assessment of smoke-free school bans^25^. All results in terms of incremental cost-effectiveness ratios (ICERs) are presented in Table 2.

**Table 2:** Incremental cost-effectiveness results of included studies

*Study* *Year*	*Location*	*Intervention*	ICER*
Donaldson et al.^28^ 2011	India	Complete smoking ban	Dominated $13/LYS (without medical treatment saved)
Højgaard et al.^27^ 2011	Denmark	Ban on smoking in enclosed public areas	$17056/LY
Higashi et al.^24^ 2011	Vietnam	Smoking bans (in public or in workplaces)	$7/DALY averted (public ban) $33/DALY averted (workplace ban) Dominated where cost offsets used
Lai et al.^29^ 2007	Estonia	Clean indoor air law	Dominated
Leão et al.^25^ 2020	EU countries	Ban on smoking in public places; school smoke-free ban	Non-school smoking bans assuming 26% and 8% mean prevalence reductions: $5–18/HLY in Germany, $26–86/HLY in Ireland, $21–68/HLY in Italy, $31–102/HLY in Belgium, $49–125/HLY in Portugal, $136–160/HLY in the Netherlands, and $115–375/HLY in Finland School smoking bans: $19/HLY in Germany, $20/HLY in Ireland, $44/HLY in the Netherlands, $53/HLY in Belgium, $67/HLY in Portugal, $136/HLY in Italy
Matheos et al.^26^ 2023	Indonesia	Ban on smoking in public places	Dominated
Ngalesoni et al.^23^ 2017	Tanzania	Smoke-free public places and smoke-free workplaces	Dominated
Nguyen et al.^22^ 2021	Vietnam	Smoke-free public places	$41/DALY
Ong and Glantz^30^ 2005	United States	Smoke-free workplace policy	$814/QALY

DALY: disability adjusted life year. EU: European Union. HLY: healthy life year. ICER: incremental cost effectiveness ratio. QALY: quality adjusted life year. LY: life years. LYS: life years saved.

* Currency standardized to 2024 US$.

### Thematic analysis

Our analysis identified smoking-ban interventions across three settings: those in public places, those in workplaces, and those in schools.

*Public places*

Four studies found smoking bans in public places to be a highly cost-effective strategy compared to the status quo^22,26-28^. Interventions included complete smoking bans, bans on smoking in enclosed areas, and bans on smoking in public places. In the Indonesian setting, a smoking ban would be the dominant strategy and save $93.8 billion in healthcare costs^26^. A study in India found that a complete smoking ban in all public places would be cost-saving when compared to a partial ban^28^. Some of these results were conservative; for example, in Denmark, healthcare cost savings related to reduced passive smoking were not included in the model, resulting in the largest ICER in our review ($17056)^27^.

*Workplaces*

One study found that workplace smoking bans resulted in positive health and economic benefits. Modeling a state-wide smoke-free workplace policy in Minnesota showed the possibility of generating 10400 quitters and an ICER of $814/QALY^30^.

Two studies assessed smoking bans in both public places and workplaces, allowing for some level of comparison^23,24^. Higashi et al.^24^ found that public bans are more cost-effective than workplace bans ($7/DALY averted vs $33/DALY averted); however, both interventions dominated the comparator of no intervention when cost-offsets were accounted for in the analysis. Results in Ngalesoni et al.^23^ identified smoke-free environment strategies in both the public and workplace as high value for money, in all instances dominating a scenario of no intervention.

*School*

A single study modelled school-based smoking bans^25^. Leão et al.^25^ found that school smoking bans were more cost-effective than non-school bans (€253/HLY vs €92/HLY). Cost-effectiveness results were sensitive to the costs of implementation, short-term effectiveness, initial smoking rates, dimensions of the target population, and weight of smoking in overall mortality and morbidity.

## DISCUSSION

Smoke-free policies are effective and often dominant strategies compared to the status quo. Nine studies analyzing the cost-effectiveness of such interventions were included in this systematic review. Studies overwhelmingly found that interventions to create smoke-free public places, workplaces, and schools were all cost-effective, and often a dominant (i.e. less costly and with better outcomes) strategy when compared with the status quo. The inclusion of healthcare costs and savings related to reductions in passive smoking resulted in further cost savings.

The evidence identified in our review often covered areas where the heaviest tobacco-related illness and death burdens are observed. Most studies in our review were conducted in low- and middle-income countries, where 80% of tobacco users worldwide reside^31^. Findings highlighted in our review are therefore contextually appropriate for settings that may benefit most significantly from smoking-ban interventions.

Smoke-free policies have already been identified as one of the most effective measures against the health and economic burden of tobacco use^32^. Our review further emphasizes the tremendous value of these strategies. Furthermore, there is considerable public support for the use of smoke-free policies in public places^33^. Through the approach and consideration of different types of smoking bans, our review corroborates the existing body of literature on the relative value of full smoking bans over partial bans. In particular, full smoking bans are associated with significant reductions in environmental tobacco smoke exposure and impact smoking prevalence, while partial bans do not^34,35^. Partial bans in many instances have not been found to be more effective than no ban.

This finding provides evidence that may favor implementing mandatory full smoking bans over partial bans. The mandatory full smoking ban characteristics may be especially relevant for reducing tobacco smoke in cities that have implemented smoke-free city-related policies. For example, the cities of Mecca and Medina in Saudi Arabia were declared smoke-free in 2002; all commercial activities related to tobacco were prohibited, though, as it was not a national ban, the personal possession of tobacco is allowed^36^. A 2007 report noted that some implementation challenges were encountered in the early stages, common to many local-level policies globally, largely due to the absence of a nationwide regulatory framework at that time^36^. Nonetheless, these early experiences offered important lessons that informed the evolution of more comprehensive tobacco control policies in Saudi Arabia in later years. The policy partially allows tobacco users to simply adopt minor adjustments to their behavior to continue to smoke. To minimize the exposure of tobacco smoke to the public requires policies that support smokers in reducing or stopping smoking.

In summary, this review suggests that smoking bans are a valuable strategy to improve population health outcomes and limit the economic burden of tobacco use. We note importantly that many of these studies are found to be less costly and yield better outcomes. As such, the interventions identified in this study logically should be more favorable policies compared to the status quo.

### Limitations

Excluding non-English publications from our review may omit relevant data from other regions or contexts. The use of academic publications may lead to an overrepresentation of favorable results as authors are less likely to publish – or to be able to publish – results of null or not cost-effective interventions (publication bias). As in most economic evaluations, the results are always highly dependent on model choices, including structure, input parameters, assumptions, and reported outcomes; as such, comparability is limited by the model choices put forth by authors of other studies. However, in our systematic review, we paid special attention to documenting all relevant model characteristics and, through the risk of bias assessment step, excluded studies that did not provide enough information for us to comprehensively evaluate the study. These steps were taken to enhance comparability and reduce the risk of ‘model hacking’ results in our study.

## CONCLUSIONS

This review summarizes peer-reviewed publications on the cost-effectiveness of smoking bans in various settings, including public spaces, workplaces, and schools. Smoke-free policies are cost-effective and dominant strategies that improve health outcomes and reduce economic burdens. Our review identified nine studies, which detailed the cost-effectiveness of smoke-free policies. We find evidence that smoking bans are not only a highly cost-effective (more expensive but more effective) strategy but also dominant in many cases (less expensive and more effective). Such findings suggest that these strategies are warranted by decision-makers considering the impact of these strategies on public health as well as the economy.

## Supplementary Material



## Data Availability

The data supporting this research can be found in the Supplementary file.
