# Supplementary material for: Cost-effectiveness of smoke-free interventions: A systematic review
Source: Tob Prev Cessat. 2025 Nov 26;11:10.18332/tpc/211801. doi: 10.18332/tpc/211801 (PMC12648411; doi:10.18332/tpc/211801)
Supplement: Supplementary file 1 [file TPC-11-52-s1.pdf]

Supplemental Materials

Appendix 1: Search Strings

**PubMed**	
**1**	Air Pollution/lj, pc
**2**	Tobacco Smoke Pollution/lj, pc
**3**	Air Pollution, Indoor/lj, pc
**4**	Smoke-Free Policy/
**5**	"clean air"[Title/Abstract:~1]
**6**	smok*[Title/Abstract] AND (ban[Title/Abstract] OR bans[Title/Abstract] OR banned[Title/Abstract] OR law[Title/Abstract] OR laws[Title/Abstract] OR policy[Title/Abstract] OR policies[Title/Abstract] OR prohibit*[Title/Abstract] OR restrict*[Title/Abstract] OR regulat*[Title/Abstract] OR legislat*[Title/Abstract] OR ordinance*[Title/Abstract])
**7**	tobacco[Title/Abstract] AND (ban[Title/Abstract] OR bans[Title/Abstract] OR banned[Title/Abstract] OR law[Title/Abstract] OR laws[Title/Abstract] OR policy[Title/Abstract] OR policies[Title/Abstract] OR prohibit*[Title/Abstract] OR restrict*[Title/Abstract] OR regulat*[Title/Abstract] OR legislat*[Title/Abstract] OR ordinance*[Title/Abstract])
**8**	#1 or #2 or #3 or #4 or #5 or #6 or #7
**9**	(Cost-benefit analysis/) AND (Cost-benefit analysis/)
**10**	cost-effectiveness analysis[Title/Abstract]
**11**	cost-benefit analysis[Title/Abstract]
**12**	economic evaluation*[Title/Abstract]
**13**	cost-utility analysis[Title/Abstract
**14**	#9 OR #10 OR #11 OR #12 OR #13
**15**	#8 AND #14
**16**	letter[pt] OR editorial[pt] OR conference review [pt]OR published erratum [pt] OR case reports [pt] OR interview[pt]
**17**	#15 NOT #16

**Web of Science**	
**1**	AB= (smoke-free policy OR air pollution OR tobacco smoke pollution)
**2**	AB= ((smok*) AND (ban OR bans OR banned OR law OR laws OR policy OR policies OR prohibi* OR restrict* OR regulat* OR legislat* OR ordinance*))
**3**	AB= ((tobacco) AND (ban OR bans OR banned OR law OR laws OR policy OR policies OR prohibi* OR restrict* OR regulat* OR legislat* OR ordinance*))
**4**	#1 OR #2 OR #3
**5**	AB=("cost-benefit analysis" OR "cost benefit analysis" OR "cost-effectiveness analysis" OR "cost effectiveness analysis" OR "economic evaluation" OR "cost-utility analysis" OR "cost utility anlaysis")
**6**	#4 AND #5

**Cochrane**

**1**	MeSH descriptor: [Air Pollution, Indoor] explode all trees
**2**	MeSH descriptor: [Air Pollution] explode all tree
**3**	MeSH descriptor: [Tobacco Smoke Pollution] explode all trees
**4**	MeSH descriptor: [Smoke-Free Policy] explode all trees
**5**	("clean" NEAR/1 "air"):ti,ab,kw
**6**	(smok* NEAR/2 (ban OR bans OR banned OR law OR laws OR policy OR policies OR prohibit* OR restrict* OR regulat* OR legislat* OR ordinance*)):ti,ab,kw
**7**	(tobacco NEAR/2 (ban OR bans OR banned OR law OR laws OR policy OR policies OR prohibit* OR restrict* OR regulat* OR legislat* OR ordinance*)):ti,ab,kw
**8**	#1 OR #2 OR #3 OR #4 OR #5 OR #6 OR #7
**9**	MeSH descriptor: [Smoking Cessation] explode all trees
**10**	MeSH descriptor: [Tobacco Use Cessation] explode all trees
**11**	(environmental tobacco smoke):ti,ab,kw
**12**	(second hand smoke OR secondhand smoke OR second-hand smoke):ti,ab,kw
**13**	(passive NEAR/3 smoking):ti,ab,kw
**14**	(smoking NEAR/3 involuntary):ti,ab,kw
**15**	(smoking cessation):ti,ab,kw
**16**	(tobacco consumption):ti,ab,kw
**17**	(smok* NEAR/2 (quit* OR stop* OR ceased OR abstain* OR abstin* OR prevent*)):ti,ab,kw
**18**	#9 OR #10 OR #11 OR #12 OR #13 OR #14 OR #15 OR #16 OR #17
**19**	MeSH descriptor: [Cost-Benefit Analysis] explode all trees
**20**	(cost-effectiveness analysis):ti,ab,kw
**21**	(cost-benefit analysis):ti,ab,kw
**22**	(economic evaluation*):ti,ab,kw
**23**	(cost-utility analysis):ti,ab,kw
**24**	#19 OR #20 OR #21 OR #22 OR #23
**25**	#8 AND #18 AND #24

Appendix 2. Quality Assessment Scoring using the Consolidated Health Economic Evaluation Reporting Standards (CHEERS) guidelines

Authors (Year)	Title	Abstract	Intro	Methods: health economic analysis plan	Methods: target pop	Methods: settings	Methods: Perspective	Methods: Comparators	Methods: Horizon	Methods: Discount Rate	Methods: Health outcomes mesaure	Methods: Valuation outcomes	Methods: Costs	Methods: Currency	Methods: Model	Methods: Assumptions	Methods: Heterogeneity	Methods: Distributional effects	Methods: Uncertainty	Methods: Engagement	Results: Parameters	Results: ICER / main results	Results: Uncertainty	Results: Engagement with patients	Discussion	Funding	COI	Overall Score
Alpert et al. (2007)*	Y	Y	Y	N	Y	Y	N	N	N	N	Y	Y	N	N	N	N	Y	N	Y	N	N	N	N	N	N	N	N	9
Donaldson et al. (2011)	Y	Y	Y	Y	Y	Y	Y	Y	Y	Y	Y	Y	Y	Y	Y	Y	N	Y	Y	N	Y	Y	Y	N	Y	N	Y	23
Higashi et al. (2011)	Y	Y	Y	Y	Y	Y	Y	Y	Y	Y	Y	Y	Y	Y	Y	Y	N	N	Y	N	Y	Y	Y	N	Y	N	N	21
Højgaard et al. (2011)	Y	Y	Y	Y	Y	Y	Y	Y	Y	Y	Y	Y	Y	Y	Y	Y	Y	N	Y	N	Y	Y	Y	N	Y	Y	N	23
Lai et al. (2007)	Y	Y	Y	Y	Y	Y	Y	Y	Y	Y	Y	Y	Y	Y	Y	Y	N	N	Y	N	Y	Y	Y	N	Y	Y	N	22
Leão et al. (2020)	Y	Y	Y	Y	Y	Y	Y	Y	Y	Y	Y	Y	Y	Y	Y	Y	N	N	Y	N	Y	Y	Y	N	Y	Y	Y	23
Matheos et al. (2023)	Y	Y	Y	Y	Y	Y	Y	Y	Y	Y	Y	Y	Y	Y	Y	Y	Y	N	Y	N	Y	Y	Y	N	Y	Y	Y	24
Ngalesoni et al. (2017)	Y	Y	Y	Y	Y	Y	Y	Y	Y	Y	Y	Y	Y	Y	Y	Y	N	N	Y	Y	Y	Y	Y	N	Y	Y	N	23
Nguyen et al. (2021)	Y	Y	Y	Y	N	Y	Y	Y	Y	Y	Y	Y	Y	Y	Y	Y	N	N	Y	N	N	Y	Y	N	Y	Y	Y	21
Ong and Glantz (2005)	Y	Y	Y	Y	Y	Y	N	Y	Y	Y	Y	Y	Y	N	Y	Y	Y	N	Y	N	Y	Y	Y	N	Y	N	N	20
Pieroni et al. (2013)*	N	N	Y	N	Y	Y	N	Y	Y	N	Y	Y	Y	N	N	Y	N	N	N	N	N	Y	N	N	N	Y	N	11

Abbreviations: COI: conflict of interest, ICER: incremental cost-effectiveness ratio, N: No, Y: Yes
**** :** excluded from review due to low-quality scores*

Appendix 3. Details of included studies

Study	Study Characteristics			Results
	Descriptive characteristics	Technical characteristics	Cost and effectiveness	
Donaldson et al. (2011)	**Perspective:** Societal **Intervention/Comparator:** Public places/Partial smoking ban **Simulated population**: Smokers in the state of Gujarat >age 20 **Country/Currency (adj. year):** India; INR (2008)	**Modeling approach:** Decision analytic model **Time horizon:** 10 year **Discounting:** 3% **Threshold used:** $880 (38,000Rs) (GDP per capita)	**Cost/source:** Direct; Non-direct / Healthcare and consumer expenditure data from the 2004 National Sample Survey (NSS) in India, WHO CHOICE **Effectiveness measure**: LYS (life years saved); AMIs averted	**Results:** When compared to the current partial ban, a complete smoking ban would save an additional 438,000 LYs based on the base case estimate that 3% of smokers would quit using cigarettes or bidis after implementation of the law. **Author's conclusions:** Implementing a complete smoking ban covering all public places throughout Gujarat would be a cost saving alternative to the current partial ban described in COTPA and the 2008 Prohibition of Smoking in Public Places Rules for reducing tobacco-related disease outcomes. **% CHEC items satisfied**: 85%
Højgaard et al. (2011)	**Perspective:** Societal **Intervention/Comparator:** Public place/No intervention **Simulated population:** Danish population **Country/Currency (adj. year):** Denmark; DKK (2011)	**Modeling approach:** Markov model **Time horizon:** 10 years and Lifetime **Discounting:** 3.5% **Threshold used:** NR	**Cost/source:** Direct; Non-direct / Literature (Reindahl) **Effectiveness measure:** LY gained	**Results:** Costs per life-year gained by a smoking ban are 40,645 to 64,462 DKK. These results are conservative as they do not include the healthcare cost saving related to reduced passive smoking. **Author's conclusions:** Results indicate that smoking ban in enclosed public places both in the short and long term is a cost-effective strategy compared with the status quo. **% CHEC items satisfied:** 78%

Higashi et al. (2011)	**Perspective:** Government **Intervention/Comparator:** Both public and workplace / **Simulated population:** Smokers as estimated from 1999 census **Country/Currency (adj. year):** Vietnam; VND (2006)	**Modeling approach:** Markov model **Time horizon:** 10 year **Discounting:** 3% **Threshold used:** 11,543,300 VND (GDP per capita)	**Cost/source:** Direct / State budget regulation, government expenses, market data **Effectiveness measure:** DALY	**Results:** The modelled interventions are all highly cost effective (without cost offset) or cost saving (with cost offset). **Author's conclusions:** Interventions to reduce the harm from tobacco use appear to be highly cost effective and should be considered as priorities in the context of Vietnam. **% CHEC items satisfied:** 85%
Lai et al. (2007)	**Perspective:** Societal **Intervention/Comparator:** /No intervention **Simulated population:** Estonian population **Country/Currency (adj. year):** Estonia; EEK (2000)	**Modeling approach:** WHO Generalized Cost Effectiveness Analysis **Time horizon:** Lifetime **Discounting:** 3% **Threshold used:** EEK 90,454 (GDP per capita)	**Cost/source:** Direct; Non-direct / Estonian Health Insurance Fund database, budgets from Ministry of Social Affairs **Effectiveness measure:** DALY	**Results:** EEK 453 per additional DALY averted **Author's conclusions:** Interventions in alcohol and tobacco control are cost-effective, and broad implementation of these interventions to upgrade current situation is warranted from the economic point of view. **% CHEC items satisfied:** 81%
Leão et al. (2020)	**Perspective:** Government **Intervention/Comparator:** Public places and schools/No modification **Simulated population:** Minors (17 years old or younger) **Country/Currency (adj. year):** EU countries; Euro (2016)	**Modeling approach:** Markov model **Time horizon**: 18 years **Discounting:** 3.5% **Threshold used:** GDP per capita (ranging from €22,500 in Portugal to €53,300 in Ireland)	**Cost/source:** Direct / Ingredients based approach using survey **Effectiveness measure:** Healthy Life Years	**Results:** Compared to the nonintervention scenario, the incremental cost-effectiveness ratio would be below €2500 per HLY for non-school bans, below €500 per HLY for school bans, and below €5000 for school education programs, even assuming the worst effectiveness scenario. **Author's conclusions:** Non-school bans cost up to €253.23 per healthy life

				year and school smoking bans up to €91.87 per healthy life year. Cost-effectiveness depended on the costs of implementation, short-term effectiveness, initial smoking rates, dimension of the target population, and weight of smoking in overall mortality and morbidity.<br>**% CHEC items satisfied:** 85%
Matheos et al. (2023)	**Perspective:** Healthcare<br>**Intervention/Comparator:** Public place/Current situation for tobacco control<br>**Simulated population:** Indonesians aged 15 to 84 year<br>**Country/Currency (adj. year):** Indonesia; USD (2020)	**Modeling approach:** Markov decision analytic model<br>**Time horizon:** Lifetime<br>**Discounting:** 3%<br>**Threshold used:** GDP per capita (ranging from €22,500 in Portugal to €53,300 in Ireland)	**Cost/source:** Direct / Literature (Leao et al 2019)<br>**Effectiveness measure:** QALY; LYS (life years saved)	**Results:** A smoking ban in public places was estimated to save US $93.8 billion in total healthcare costs, making it a dominant strategy compared with the current situation.<br>**Author's conclusions:** In Indonesia, tobacco control measures, such as a ban on smoking in public places, are likely to be highly cost-effective and even cost saving from the healthcare system's perspective<br>**% CHEC items satisfied:** 88%
Ngalesoni et al. (2017)	**Perspective:** Government<br>**Intervention/Comparator:** Workplace and public place/No intervention<br>**Simulated population:** Tanzanian population<br>**Country/Currency (adj. year):** Tanzania; USD (2013)	**Modeling approach:** Markov model<br>**Time horizon:** 10 year<br>**Discounting:** 3%<br>**Threshold used:** $910 (GDP per capita)	**Cost/source:** Direct / Primary data from costing analysis from July 2011-June 2012<br>**Effectiveness measure:** DALY	**Results:** The modelled interventions are all very cost-effective as they fall below one times the GDP per capita for Tanzania for 2013.<br>**Author's conclusions:** The model results show that population-based tobacco control strategies such as smoke free environments offer a good value for money in the primary prevention of CVD in Tanzania.<br>**% CHEC items satisfied:** 85%

Nguyen et al. (2021)	**Perspective:** Healthcare<br>**Intervention/Comparator:** Public place/No intervention<br>**Simulated population:** Vietnamese population<br>**Country/Currency (adj. year):** Vietnam; VND (2015)	**Modeling approach:** Markov decision analytic model<br>**Time horizon:** 10 year<br>**Discounting:** 3%<br>**Threshold used:** $2,109 (GDP per capita)	**Cost/source:** Direct / Activity based costing<br>**Effectiveness measure:** DALY	**Results:** Findings demonstrate that all tobacco control interventions in this study were highly cost-effective as compared with the set threshold of one time GDP per capita.<br>**Author's conclusions:** The results from this study provide a robust message that calls for increased attention and efforts in developing an appropriate policy agenda, which jointly integrates both political and community-based interventions, to maximize intervention impact on tobacco use.<br>**% CHEC items satisfied:** 77%
Ong and Glantz (2005)	**Perspective:** NR<br>**Intervention/Comparator:** Workplace/<br>Simulated population: Minnesota's smoking populations<br>**Country/Currency (adj. year):** United States; USD (2002)	Modeling approach: Decision Tree<br>**Time horizon:** 1 year<br>**Discounting:** 3%<br>**Threshold used:** $50,000/QALY	**Cost/source:** Direct / Estimates from other states extrapolated on the basis of per-capita costs.<br>**Effectiveness measure:** QALY	**Results:** Implementing a statewide smoke-free workplace policy generated 10,400 quitters at a total cost of $8.3 million, or a cost per quitter of $799 . The equivalent cost per QALY was $506.<br>**Author's conclusions:** Implementing smoke-free workplace policies was more cost-effective than the alternative free NRT program. Smoke-free work-places are a more cost-effective method for reducing smoking, suggesting that smoke-free workplace campaigns should be a priority for public health programs, even when the primary goal is to help people stop smoking.

% CHEC items satisfied: 74%

Abbreviations: COTPA: Cigarettes and Other Tobacco Products Act, CHEC: Consolidated Health Economic Checklist, CVD: cardiovascular disease DALY: disability adjusted life year, DKK: Danish Kroner, EEK: Estonian Kroon, GDP: gross domestic product, HLY: healthy life year, INR: Indian Rupee, LY: life years, NR: not reported, NRT, QALY: quality adjusted life year, USD: United States Dollar, VND: Vietnamese Dong WHO CHOICE: World Health Organization CHOosing Interventions that are Cost-Effective

© 2025 Werner K. et al.
